# Transfusion Patterns in All Patients Admitted to the Intensive Care Unit and in Those Who Die in Hospital: A Descriptive Analysis

**DOI:** 10.1371/journal.pone.0138427

**Published:** 2015-09-17

**Authors:** Nadine Shehata, Alan J. Forster, Nadine Lawrence, Robin Ducharme, Dean A. Fergusson, Michaël Chassé, Deanna M. Rothwell, Paul C. Hébert, Alan T. Tinmouth, Kumanan Wilson

**Affiliations:** 1 Department of Medicine, Mount Sinai Hospital, University of Toronto, Toronto, Canada; 2 Department of Pathology and Laboratory Medicine, Mount Sinai Hospital, University of Toronto, Toronto, Canada; 3 Li Ka Shing Knowledge Institute, St. Michael’s Hospital, Toronto, Canada; 4 Central Ontario Region, Canadian Blood Services, Toronto, Canada; 5 Clinical Epidemiology Program, Ottawa Hospital Research Institute, Ottawa, Canada; 6 Institute for Clinical Evaluative Sciences, Ottawa, Canada; 7 Department of Medicine, University of Ottawa, Ottawa, Canada; 8 Department of Epidemiology and Community Medicine, University of Ottawa, Ottawa, Canada; 9 Department of Surgery, University of Ottawa, Ottawa, Canada; 10 Department of Medicine, University of Montreal, Montreal, Canada; University Hospital of Salamanca, SPAIN

## Abstract

While it is known that the use of health care resources increases at the end of life in patients admitted to the Intensive Care Unit (ICU), the allocation of blood products at the end of life has not been described. The objective of this study was to describe overall transfusion patterns in the ICU, and specifically in patients who die in hospital. We conducted a retrospective cohort study of adult patients admitted to the ICU of a university-affiliated hospital, who were discharged or died between November 1, 2006 and June 30, 2012. During the study period, 10,642 patients were admitted at least once to the ICU. Of these patients, 4079 (38.3%) received red blood cells (RBCs), plasma or platelets in the ICU. The ICU mortality rate was 28.1% and in-hospital mortality rate was 32.3%. Among 39,591 blood product units transfused over the course of the study in the ICU (18,144 RBC units, 16,920 plasma units and 4527 platelet units), 46.2% were administered to patients who later died within the same hospitalization (41.2% of RBCs, 50.4% of plasma and 50.8% of platelets). Of all blood product units (RBCs, plasma and platelets) administered in the ICU over the study period, 11% were given within the last 24 hours before death. A large proportion of blood products used in the ICU are administered to patients who ultimately succumb to their illness in hospital, and many of these blood units are given in close proximity to death.

## Introduction

A considerable proportion of patients in critical care units are transfused. Thirty to 45% receive red cell transfusion [1,2], 10–30% receive plasma transfusion [3,4] and 10–20% receive platelets [5,6]. Older age, the need for emergent admission, as well admissions for trauma and surgery have been associated with increased red cell transfusion [1]. Plasma is predominantly used to treat bleeding patients [4] whereas platelets are transfused prophylactically [7,8].

Increased use of health care resources at the end of life has been well described [5, 9, 10]. Blood product requirements as part of life sustaining treatment at the end of life have not been described. Like other medical therapies, such as the need for critical care services [9], blood product use may increase in individuals who are at higher risk of death. To assess the extent of blood product use in patients who ultimately die, the ICU population is particularly appropriate given the high acuity, high mortality rate and high blood product utilization. The primary objective of this study was to describe the utilization of blood products in patients admitted to the intensive care unit (ICU) and the transfusion rates of ICU patients who ultimately die.

## Materials and Methods

### Data Sources

Data was obtained from the Ottawa Hospital’s Data Warehouse in Ottawa, Canada. The Data Warehouse is a data repository from the hospital’s operational information systems. These systems include patient registration, the discharge abstract database, laboratory, pharmacy and radiology results from 2006 onward. We used the Ottawa Hospital’s Data Warehouse to obtain all data on patient characteristics, procedures and primary diagnoses, blood components transfused (i.e. plasma, platelets, and red blood cells (RBCs)), length of stay, in-hospital all-cause mortality and in-ICU all-cause mortality. This administrative database has been used to evaluate health-related questions in several previous studies [6, 11–14]. Ethics approval for this study was obtained from the Ottawa Health Science Network Research Ethics Board (#20120280-01H). Patient information in the data repository was anonymized and de-identified prior to analysis. As per the institutional ethics policy, this data was used for the approved study without express informed consent from study subjects.

### Study Design

We conducted a retrospective cohort study of adult patients in the ICU. Patients admitted to one of two 30-bed medical/surgical academic ICUs within The Ottawa Hospital and discharged between November 1, 2006 and June 30, 2012 were included. Our study cohort included patients admitted to the ICU at any time during their hospital stay, and those awaiting intensive care beds. Cardiac surgery patients were not included in the ICU cohort as The Ottawa Hospital has a separate cardiac centre. The Ottawa Hospital also has a “step-down unit” for lower acuity patients. In order to ensure that all deaths in our study cohort were captured; we focused our analysis on the last admission for patients having multiple qualifying hospital admissions during the study period. We included all qualifying admissions in descriptions of overall blood product utilization. The Ottawa Hospital serves 1.2 million residents of Ottawa and Eastern Ontario. The ICUs are supervised by academic critical care staff with a full complement of fellows and residents.

We identified patients who were admitted to the ICU at any time during their hospital stay, and determined: 1) how many of these patients received blood transfusions (RBCs, plasma or platelets) while under the ICU service, 2) how many of these patients subsequently died from any cause either in the ICU or in hospital, and 3) the amount of blood products administered to these patients during their ICU stay. We also determined the number of blood product units administered in the ICU in the days leading up to death or discharge from hospital.

For patients having multiple ICU stays within one hospitalization, all of their time spent in ICU was grouped together and the total length of stay included all days spent in ICU, either consecutively or not.

It was not possible from our data to compute physiology-based ICU acuity scores. We therefore reported baseline characteristics and calculated the Charlson index, which have been shown, when taken together, to be as predictive of ICU death as commonly used ICU acuity scores such as the SOFA or APACHE II [15]. We used the International Classification of Diseases 10 codes [16, 17] and the revised Schneeweiss weights [18] to calculate the Charlson index.

### Blood Product Distribution in the ICU

To describe how blood products were distributed over the course of patients’ ICU stays, we identified the date of each patient’s death or discharge, and calculated the amount of each type of blood product received in the ICU on each of the 7 days leading up to death or discharge. We computed proportions of patients transfused on each day using denominators that included all patients who were in the ICU on that day leading up to death or discharge. On any given day (in relation to death or discharge), a patient was counted if s/he were in the ICU on that day. The percentage of total blood product volume administered on each day in relation to death or discharge was calculated as a percentage of the total number of units used in the ICU over the study period. Further, we identified patients who underwent a massive transfusion on the last day before death or discharge as those who received at least 4 units of RBCs within one hour.

### Statistical Analyses

All analyses were conducted at the level of the patient. Data for continuous variables are presented as means (with standard deviation) or medians (with Interquartile Ranges (IQR)) if the data were heavily skewed. Data for categorical variables are presented as frequencies and percentages.

To test for significant transfusion trends in transfused patients who died in hospital or were discharged alive, we built logistic regression models of the proportion of patients transfused each type of blood product on the 7 days leading up to death or discharge. We used Poisson regression to model the mean number of blood product units transfused over the 7 days leading up to death or discharge, and to model the proportion of the total number of blood product units transfused in the ICU over the 7 days leading up to death or discharge. We also built logistic regression models to test for trends in the proportions of hospitalized patients who received RBCs, platelets and plasma over the years of study, each for patients discharged alive and patients who died in hospital. Results from logistic regression models are presented as Odds Ratios (OR) and 95% confidence intervals (95% CI). Results from Poisson regression are presented as Rate Ratios (RR) and 95% CI.

## Results

### Demographics

There were a total of 10,642 patients admitted to the ICU within the study period (Table 1). During the same interval there were 154,391 patients admitted to hospital. The median (IQR) patient age was 64 (51–75), and 56.7% of patients were male. The median (IQR) ICU length of stay was 4 (2–10) days, and the median (IQR) overall hospital length of stay was 11 (5–26) days. Patients who died in hospital had shorter median ICU and hospital lengths of stay than patients who were discharged alive. Overall, nearly 65.8% (n = 7001) of patients were mechanically ventilated during their ICU stay, ranging from 59.8% for patients who did not receive any blood products to 78.4% for patients who received platelet transfusions. Nearly 25% of transfused patients received renal replacement therapy, compared to 6% for non-transfused patients. The median Charlson score was 2 across all group and 25% of patients had a Charlson index above 5.

**Table 1:** Characteristics of patients admitted to the ICU.

	Overall N = 10642	Received RBCs^a^ N = 3477	Received plasma^a^ N = 1967	Received platelets^a^ N = 1058	Did not receive blood products^b^ N = 6563
**Median (IQR) patient age at admission (yrs)**	64 (51–75)	65 (53–75)	65 (52–76)	62 (51–72)	64 (50–75)
**Male, N (%)**	6032 (56.7)	1948 (56.0)	1183 (60.1)	655 (61.9)	3718 (56.7)
**Median (IQR) Charlson Score**	2 (0–5)	2 (1–5)	2 (1–6)	2 (1–5)	2 (0–4)
**Charlson Index, N (%)**					
0	2671 (25.1)	764 (22.0)	444 (22.6)	207 (19.6)	1774 (27.0)
1–2	3426 (32.2)	999 (28.7)	549 (27.9)	371 (35.1)	2253 (34.3)
3–4	1879 (17.7)	636 (18.3)	338 (17.2)	193 (18.2)	1129 (17.2)
5+	2666 (25.1)	1078 (31.0)	636 (32.3)	287 (27.1)	1407 (21.4)
**Surgical patient, N (%)**	3866 (36.3)	1473 (42.4)	756 (38.4)	328 (31.0)	2192 (33.4)
**Marital Status**					
Divorced (N = 568), n (%)	n/a	197 (34.7)	114 (20.1)	59 (10.4)	342 (60.2)
Married/Common law (N = 5742), n (%)	n/a	2000 (34.8)	1107 (19.3)	635 (11.1)	3411 (59.4)
Separated (N = 240), n (%)	n/a	60 (25.0)	31 (12.9)	26 (10.8)	171 (71.3)
Single (N = 1889), n (%)	n/a	572 (30.3)	330 (17.5)	171 (9.0)	1224 (64.8)
Unknown (N = 938), n (%)	n/a	217 (23.1)	176 (18.8)	72 (7.7)	652 (60.3)
Widowed (N = 1265), n (%)	n/a	431 (34.1)	209 (16.5)	95 (7.5)	763 (60.3)
**Mechanical ventilation, N (%)**	7001 (65.8)	2659 (76.5)	1539 (78.2)	830 (78.4)	3926 (59.8)
**Renal replacement therapy, N (%)**	1215 (11.4)	767 (22.1)	472 (24.0)	249 (23.5)	380 (5.8)
**Primary diagnosis category, N (%)**					
Cancer	1132 (10.6)	470 (13.5)	235 (11.9)	219 (20.7)	578 (8.8)
Cardiovascular diseases	2357 (22.1)	562 (16.2)	324 (16.5)	156 (14.7)	1681 (25.6)
Endocrinology including diabetes	260 (2.4)	76 (2.2)	34 (1.7)	15 (1.4)	174 (2.7)
Gastrointestinal and liver disease	1042 (9.8)	513 (14.8)	383 (19.5)	162 (15.3)	446 (6.8)
Infectious diseases	1195 (11.2)	512 (14.7)	332 (16.9)	186 (17.6)	570 (8.7)
Others	1429 (13.4)	365 (10.5)	222 (11.3)	95 (9.0)	992 (15.1)
Respiratory diseases	1429 (13.4)	368 (10.6)	144 (7.3)	79 (7.5)	1001 (15.3)
Trauma and poisoning	1798 (16.9)	611 (17.6)	293 (14.9)	146 (13.8)	1121 (17.1)
**Overall**					
Median (IQR) ICU length of stay (days)	4 (2–10)	9 (4–17)	7 (2–15)	8 (3–16)	3 (1–7)
Median (IQR) hospital length of stay (days)	11 (5–26)	20 (9–42)	15 (6–34)	17 (7–41)	8 (3–18)
**Died in hospital (N = 3434)**					
Median (IQR) ICU length of stay (days)	3 (1–9)	7 (2–16)	4 (1–13)	6 (1–14)	2 (1–5)
Median (IQR) hospital length of stay (days)	7 (2–17)	13 (5–28)	9 (2–22)	11 (4–24)	4 (1–11)
**Discharged alive (N = 7208)**					
Median (IQR) ICU length of stay (days)	5 (2–10)	10 (4–18)	8 (4–17)	10 (4–18)	4 (2–7)
Median (IQR) hospital length of stay (days)	13 (6–29)	27 (13–49)	23 (11–44)	27 (13–51)	10 (5–21)

IQR: interquartile range; RBCs: red blood cells

^a^ Categories are not mutually exclusive. Patients who received more than one type of blood product are included under multiple categories.

^b^ Patients who did not receive RBCs, platelets, or plasma.

### Transfusions in the ICU

At least one type of blood product was administered in the ICU to 4079 (38.3%) patients during their stay. RBCs were administered to 3477 (32.7%) patients and plasma and platelets were administered to 1967 (18.5%) and 1058 (9.9%) patients, respectively. RBCs, plasma and platelets were transfused to 40%, 28% and 16% respectively of ICU patients who died within their hospitalization, compared to 34%, 16% and 11% of all patients under any service who died in the hospital (data not shown). Overall, patients who received blood products were older and had longer lengths of stay as compared to patients who did not receive blood products. A higher proportion of patients who did not receive blood products had lower Charlson comorbidity scores (indicating low burden of disease), compared to patients who received blood products (Table 1). Of patients who received RBCs, plasma or platelets, 31.0%, 32.3% and 27.1% respectively had the highest Charlson score (5 or more), compared to 21.4% who did not receive blood.

### Mortality rates and blood product use

The overall ICU mortality rate was 28.1% and in-hospital mortality rate was 32.3%. The in-hospital mortality rate ranged from a low of 27.3% to a high of 50.6% and the ICU mortality rate ranged from a low of 23.7% to a high of 45.8%, for patients who did not receive blood products and for patients who received platelets, respectively (Table 2).

**Table 2:** In-hospital mortality rates and ICU mortality rates by type of blood product.

	Overall N = 10642	Received RBCs^a^ N = 3477	Received plasma^a^ N = 1967	Received platelets^a^ N = 1058	Did not receive blood products^b^ N = 6563
In-hospital mortality, N (%)	3434 (32.3)	1359 (39.1)	972 (49.4)	535 (50.6)	1793 (27.3)
ICU Mortality, N (%)	2993 (28.1)	1192 (34.3)	880 (44.7)	484 (45.8)	1553 (23.7)

RBCs: red blood cells

^a^ Categories are not mutually exclusive. Patients who received more than one type of blood product can be included under multiple categories.

^b^ Patients who did not receive RBCs, platelets, or plasma.

**Fig 1:**
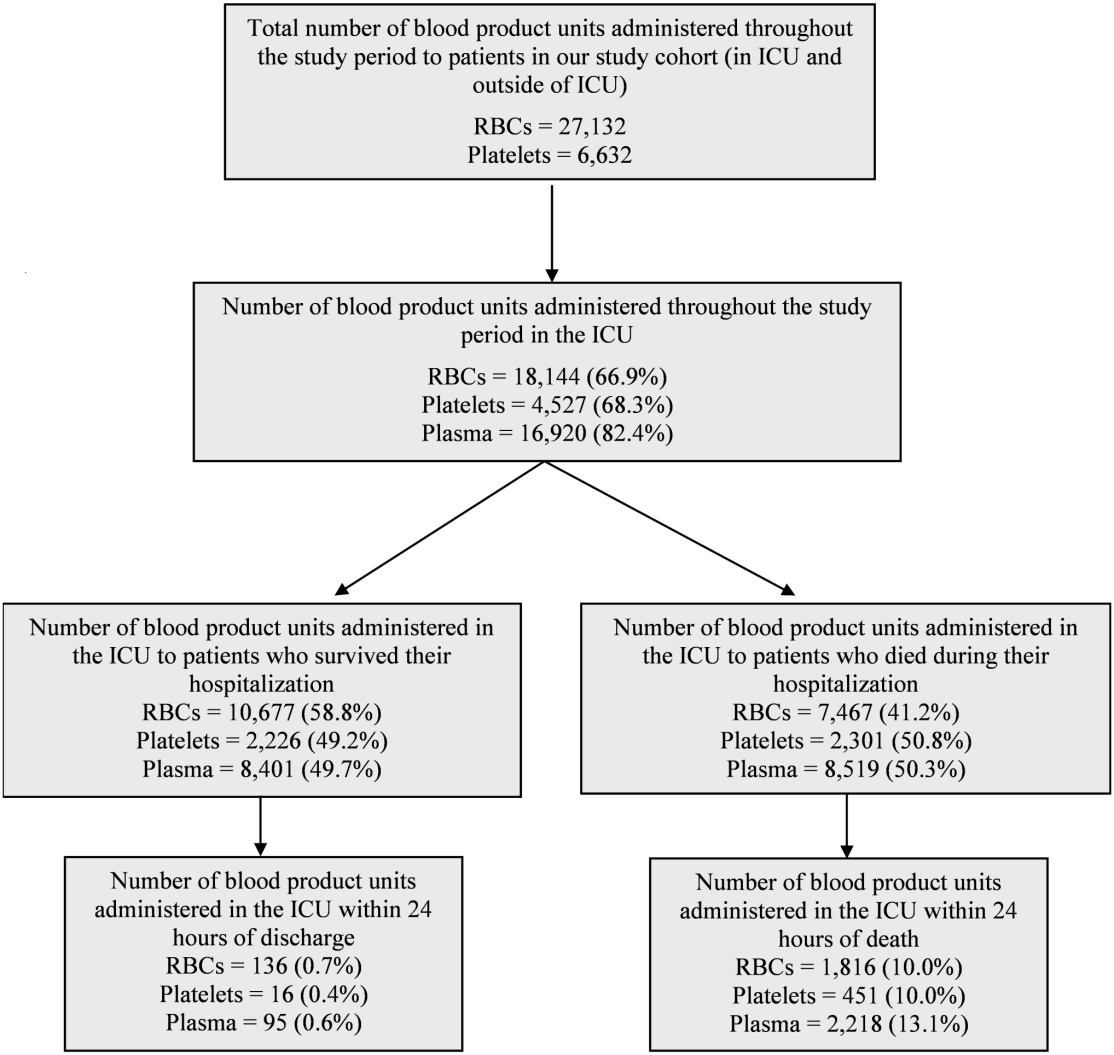
Distribution of blood products throughout the study period.

Overall, of all RBC, plasma and platelet units administered in the ICU throughout the study period, 46.2% were administered to patients who later died within the same hospitalization (41.2% of RBCs, 50.8% of platelets, 50.3% of plasma) (Fig 1). In comparison, of all blood products administered to patients hospitalized under any service during the study period, 22.9% (19.1% of RBCs, 25.9% plasma and 30.2% of platelets) were administered to those who later died during their hospitalization (data not shown). Of all blood products administered in the ICU, 10.0% of RBCs, 10.0% of platelets and 13.1% of plasma were given within 24 hours of death.

### Blood Product Distribution in relation to discharge or death

#### Proportion of patients transfused

**Fig 2:**
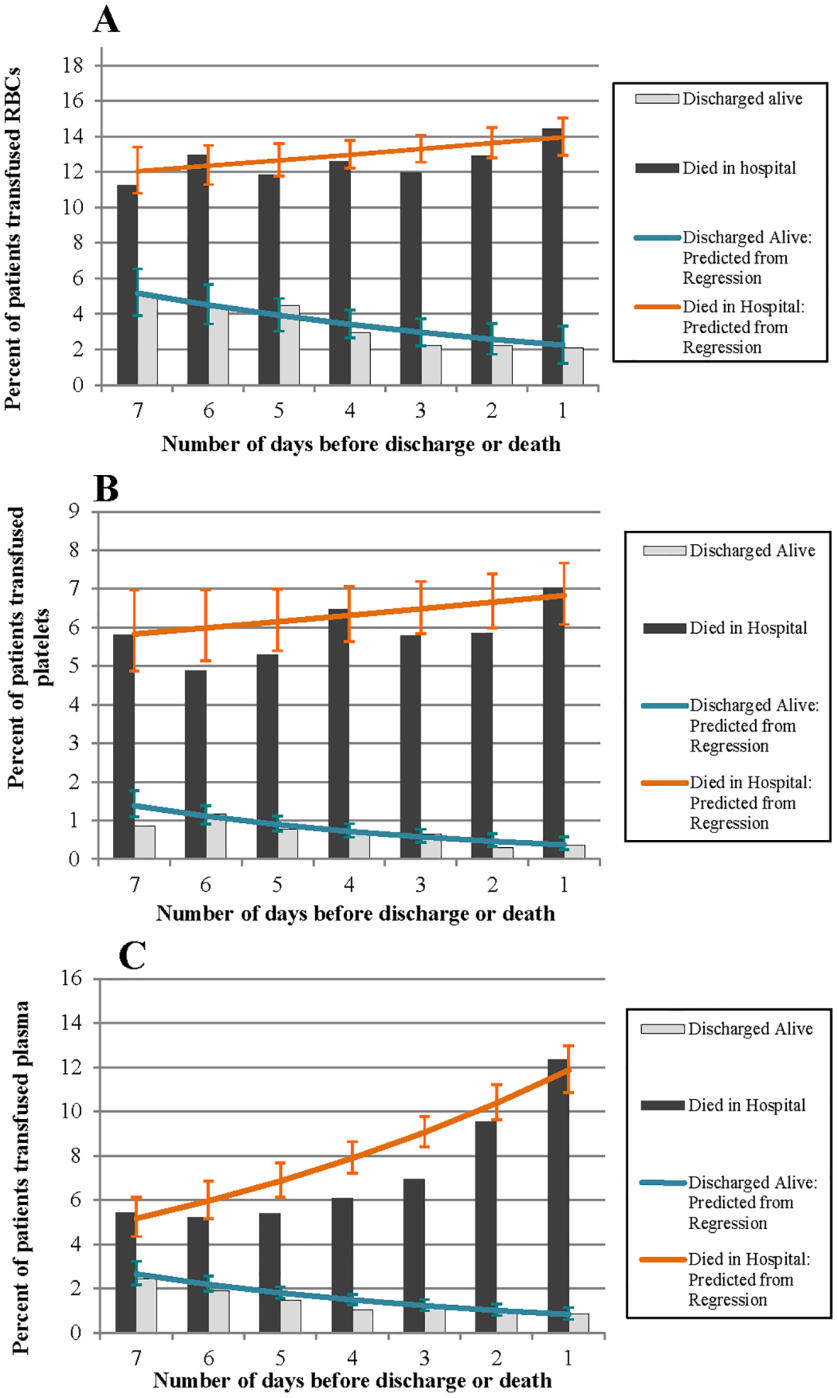
Observed and predicted percentage of patients in the ICU who were transfused A) RBCs, B) platelets and C) plasma on each of the 7 days leading up to discharge (alive) or death.

**Fig 3:**
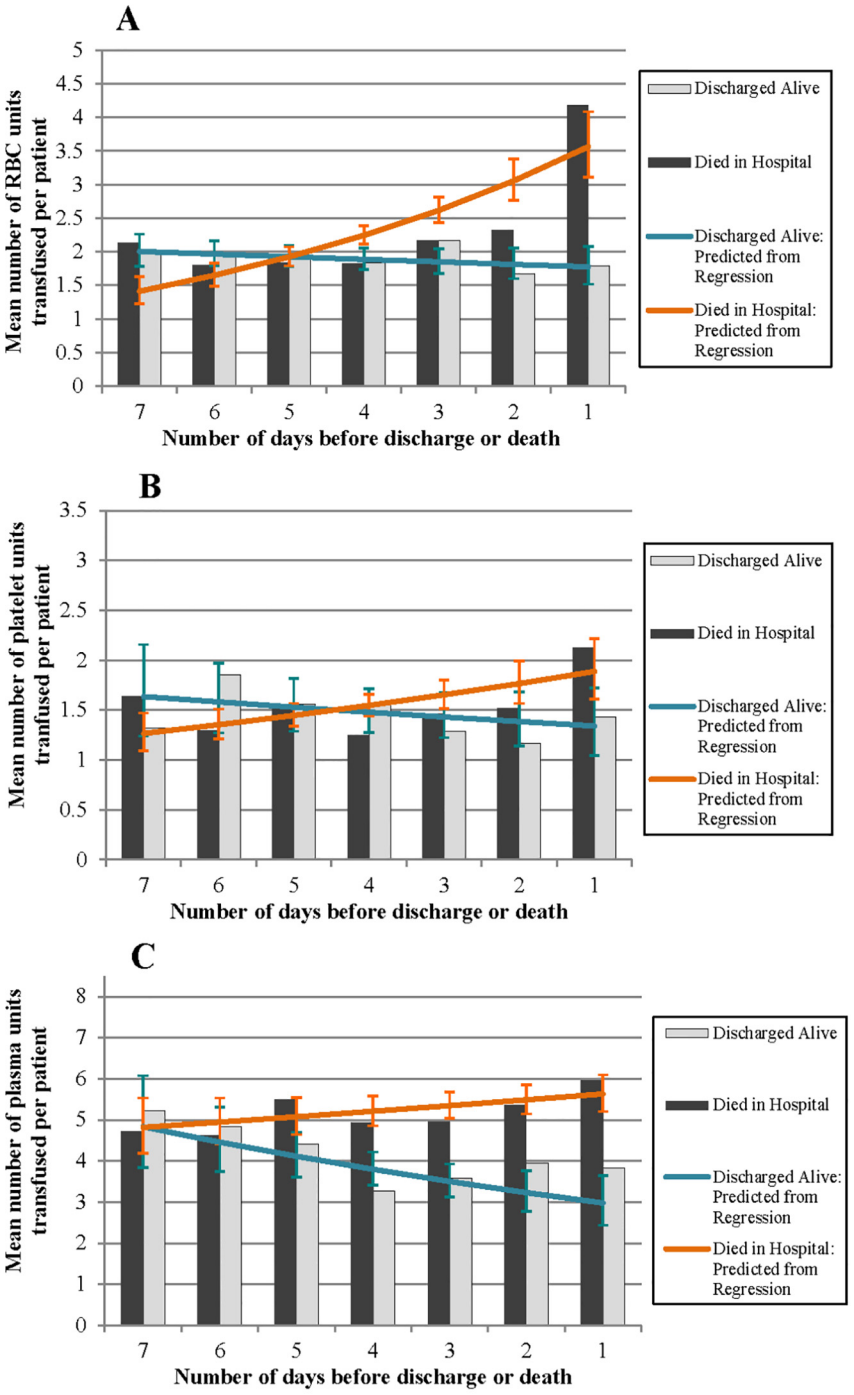
Observed and predicted mean number of A) RBC units, B) platelets units and C) plasma units administered to each transfused patient in the ICU 7 days leading up to discharge (alive) or death.

**Fig 4:**
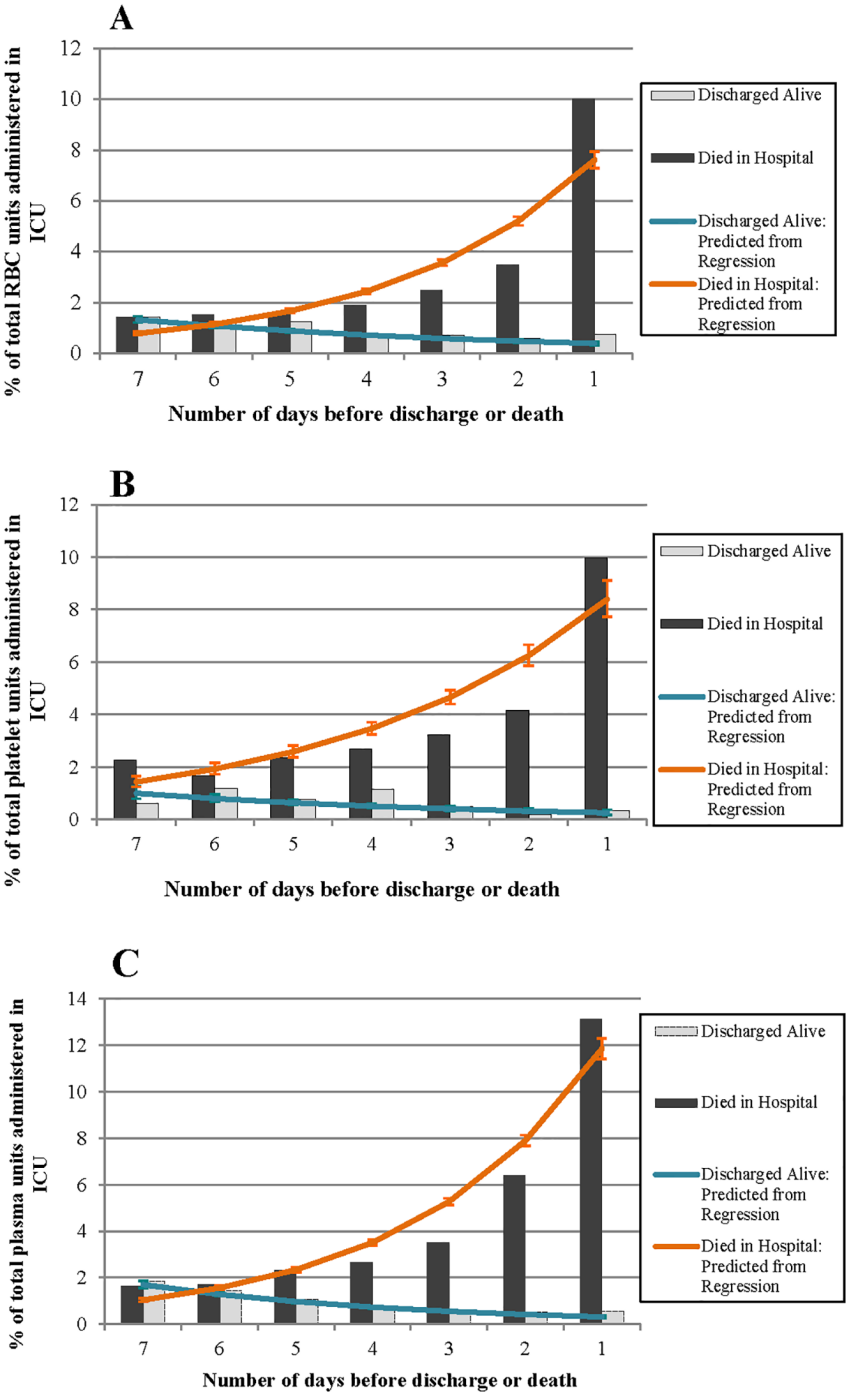
Observed and predicted percent of the total A) RBC units, B) platelets units and C) plasma units administered in the ICU on each of the 7 days leading up to discharge (alive) or death.

Figs 2 to 4 illustrate the percentage of patients in the ICU who received RBCs, plasma and platelets, the mean number of units received, and the percentage of the total volume of each blood product administered in the ICU on each of the 7 days leading up to discharge or death. The vertical bars in each figure represent the observed values, and the lines represent the predicted values based on the regression models.

In patients discharged alive, the proportion transfused RBCs, platelets and plasma significantly decreased on each day leading up to discharge (RBCs: OR (95% CI): 0.87 (0.83, 0.91); platelets: 0.82 (0.75, 0.90); plasma: 0.83 (0.77, 0.89)) (Fig 2A–2C). In patients who died, the proportion transfused RBCs or plasma significantly increased in the days leading up to death (RBCs: 1.03 (1.00, 1.06); plasma: 1.14 (1.11, 1.18)). There was no significant change in the proportion of patients transfused platelets in the days leading up to death (platelets: 1.02 (0.98, 1.05)).

Of the 435 patients who received RBCs within 24 hours of death, 37 (8.5%) underwent a massive transfusion. These patients received a mean (SD) of 20.5 (16.4) and a median (IQR) of 16 (12–20) units of RBCs within 24 hours of death. No patient who received RBCs within 24 hours of being discharged alive underwent a massive transfusion.

### Mean number of blood product units

For patients discharged alive, there was no significant change in the mean number of RBC and platelet units received on each day (RBCs: RR (95% CI): 0.98 (0.94, 1.01); platelets: 0.96 (0.89, 1.04)) (Fig 3A and 3B). There was a significant decrease in the mean number of plasma units transfused leading up to discharge (plasma: 0.87 (0.83, 0.91)) (Fig 3C). In patients who died, the mean number of blood product units transfused per patient significantly increased leading up to death, with the largest increase observed for RBCs (RBCs: 1.17 (1.12, 1.22); platelets: 1.07 (1.02, 1.12); plasma: 1.03 (1.00, 1.06)) (Fig 3A–3C).

### Percent of total blood product units used in the ICU

The percentage of total number of units for each blood product used in the ICU significantly decreased leading up to discharge, in patients who were discharged alive (RBCs: RR (95% CI): 0.82 (0.79, 0.84); platelets: 0.80 (0.74, 0.87); plasma: 0.75 (0.73, 0.78)) (Fig 4A–4C). In patients who died, the percentage of blood product used in the ICU prior to death significantly increased leading up to the day of death (RBCs: 1.46 (1.44, 1.49); platelets: 1.34 (1.30, 1.39); plasma: 1.50 (1.48, 1.53)) (Fig 4A–4C).

### Proportion of patients transfused over the years of study

Over the years of study, we observed a significant decrease in the proportion of hospitalized patients receiving RBC products in those who died, but not in those who were discharged alive (died in hospital: OR (95% CI): 0.94 (0.90, 0.98); discharged alive: 1.00 (0.97, 1.03)). We also observed a significant reduction over time in the proportion transfused plasma in patients who died and in those who were discharged alive (died in hospital: 0.90 (0.85, 0.94); discharged alive: 0.91 (0.88, 0.95)). There was no significant change over time in platelet use (died in hospital: 1.00 (0.94, 1.06); discharged alive: 1.03 (0.97, 1.08)).

## Discussion

In this study, we described patterns of transfusion in patients admitted to the ICU, and further explored transfusion patterns in patients who died in hospital. Nearly 40% of the patients in our study cohort received at least one type of blood product while in the ICU. Of all blood products used in the ICU over the study period 46.2% were administered to patients who died during their hospitalization and 11% were administered to patients within 24 hours of death. In patients who died, the intensity of transfusion increased in the days preceding death. Of all of the blood products, red blood cells were administered to the largest number of patients.

Our finding that a large proportion of patients who receive blood products subsequently die in hospital was somewhat expected given the higher burden of illness in patients who receive transfusions [19]. In addition the intensity of transfusion in the days preceding death identifies a proportion of critical care patients with a higher mortality rate. This was consistent with our observation of higher Charlson scores in patients requiring transfusion. Nonetheless, the observation that in some instances the mortality rate in transfused patients approached 50% advocates for a closer examination of the benefit of transfusion in these circumstances.

The high proportion of blood products administered immediately preceding death is similar to the increased use of other resources such as critical care services that occur at the end of life [5, 9]. However, this has not been previously described for blood products. While our analysis does not imply that transfusions near the end of life caused death, increased resource utilization near the end-of-life, in other settings, has not been shown to be associated with improved care and may result in a worse quality of death [20]. Could transfusion be also associated with a poor quality of death?

There are a few limitations to this study. Generalizability may be limited as this is a single center study in a health system where blood products are provided free of charge to hospitals. However, the large sample size of this study, and the inclusion of two hospital sites supports the validity of the results. The higher mortality rate observed in this study compared to that reported in the ICU literature [15] may be potentially explained by the exclusion of cardiac surgery patients who have a low mortality rate (4 to 6%) [21]. This high acuity is supported by the advanced age (median 64, IQR (51–75)), high proportion of mechanically ventilated patients (65.8%) and the proportion receiving renal replacement therapy (11.9%), suggesting that we succeeded to select the patients at the highest risk of mortality. In addition, using administrative data does not lend to an understanding of the necessity or indication for transfusion. As it has been shown that diagnoses coded in the ICU have limited validity [22], we were cautious to not rely on diagnosis codes to identify indications for transfusion. Although APACHE II scores may have been a more appropriate measure of disease severity in our study population than the Charlson score, we did not have the ability to calculate APACHE II scores using the administrative data. However, given the fact that the Charlson score, in addition to selected administrative data have been previously shown to be as good as SAPS II, SAPS III and Apache II to predict mortality in the ICU population [15], we believe that the data reported provides clinically relevant information that could be used to estimate the mortality risk of our population. Finally, the marked increase in the number of patients who received blood products prior to death is unlikely to be indicative of hemorrhage as massive hemorrhage occurs in a small proportion of the population [23], and only a small proportion of admissions to the critical service are secondary to hemorrhage.

## Conclusions

Nearly 40% of patients received at least one type of blood product while in the ICU. The most widely used blood product was red blood cells, administered to 32.6% of patients. A high percentage of admissions where transfusion was administered subsequently ended in death, and many of these transfusions happened on the day prior to death. These results should stimulate further evaluation of the benefit for transfusion in the last hours of life.

## Abbreviations:

ICU: Intensive Care Unit
RBCs: Red Blood Cells
IQR: Interquartile Range

## References

[1] Vincent JL 1, Baron JF, Reinhart K, Gattinoni L, Thijs L, Webb A, et al Anemia and blood transfusion in critically ill patients. JAMA. 2002 9 25;288(12):1499–507. 12243637 10.1001/jama.288.12.1499

[2] Corwin HL, Gettinger A, Pearl RG, Fink MP, Levy MM, Abraham E, et al The CRIT Study: Anemia and blood transfusion in the critically ill—current clinical practice in the United States. Crit Care Med 2004 1;32(1):39–52. 14707558 10.1097/01.CCM.0000104112.34142.79

[3] Lauzier F 1, Cook D, Griffith L, Upton J, Crowther M. Fresh frozen plasma transfusion in critically ill patients. Crit Care Med. 2007 7;35(7):1655–9. 17522577 10.1097/01.CCM.0000269370.59214.97

[4] Stanworth SJ, Walsh TS, Prescott RJ, Lee RJ, Watson DM, Wyncoll D, et al A national study of plasma use in critical care: clinical indications, dose and effect on prothrombin time. Crit Care. 2011;15(2):R108 10.1186/cc10129 21466676 PMC3219386

[5] Unroe KT, Greiner MA, Hernandez AF, Whellan DJ, Kaul P, Schulman KA, et al Resource use in the last 6 months of life among medicare beneficiaries with heart failure, 2000–2007. Arch Intern Med. 2011 2 14;171(3):196–203. 10.1001/archinternmed.2010.371 20937916

[6] Shehata N, Forster AJ, Naglie G, Mazer D, Tu J. Rubens F, et al Does anemia impact hospital readmissions following coronary artery bypass surgery? Transfusion. 2013 8;53(8):1688–97. 10.1111/trf.12007 23228115

[7] Arnold DM, Crowther MA, Cook RJ, Sigouin C, Heddle NM, Molnar L, et al Utilization of platelet transfusions in the intensive care unit: indications, transfusion triggers, and platelet count responses. Transfusion. 2006 8;46(8):1286–91. 16934061 10.1111/j.1537-2995.2006.00892.x

[8] Stanworth SJ, Walsh TS, Prescott RJ, Lee RJ, Watson DM, Wyncoll DL, et al Thrombocytopenia and platelet transfusion in UK critical care: a multicenter observational study. Transfusion. 2013 5;53(5):1050–8. 10.1111/j.1537-2995.2012.03866.x 22928908

[9] Teno JM, Gozalo PL, Bynum JP, Leland NE, Miller SC, Morden NE, et al Change in end-of-life care for Medicare beneficiaries: site of death, place of care, and health care transitions in 2000, 2005, and 2009. JAMA. 2013 2 6;309(5):470–7. 10.1001/jama.2012.207624 23385273 PMC3674823

[10] Reuben DB, Cassel CK. Physician stewardship of health care in an era of finite resources. JAMA. 2011 7 27;306(4):430–1. 10.1001/jama.2011.999 21791692

[11] Forster AJ, Taljaard M, Oake N, Wilson K, Roth V, van Walraven C. The effect of hospital-acquired Clostridium difficile infection on hospital length of stay. CMAJ. 2012 1;184(1): 37–42. 10.1503/cmaj.110543 22143235 PMC3255231

[12] Reimche L, Forster AJ, van Walraven C. Incidence and contributors to drug-drug interactions in hospitalized patients. J Clin Pharmacol. 2011 7;51(7):1043–50. 10.1177/0091270010378858 20926752

[13] van Walraven C, Jennings A, Wong J, Forster AJ. The influence of house-staff experience on teaching hospital mortality: the ‘July Phenomenon’ revisited. J Hosp Med. 2011 9;6(7):389–94. 10.1002/jhm.917 21916000

[14] van Klei WA, Bryson GL, Yang H, Forster AJ. Effect of Beta-blocker Prescription on the Incidence of Postoperative Myocardial Infarction after Hip and Knee Arthroplasty. Anesthesiology. 2009 10;111(4):717–24. 10.1097/ALN.0b013e3181b6a761 19707119

[15] Christensen S, Johansen MB, Christiansen CF, Jensen R, Lemeshow S. Comparison of Charlson comorbidity index with SAPS and APACHE scores for prediction of mortality following intensive care. Clin Epidemiol. 2011;3:203–211. 10.2147/CLEP.S20247 21750629 PMC3130905

[16] Deyo RA, Cherkin DC, Ciol MA. Adapting a Clinical Comorbidity Index for use with ICD 9-CM administrative databases. J Clin Epidemiol. 1992 6;45(6):613–9. 1607900 10.1016/0895-4356(92)90133-8

[17] Quan H, Sundararajan V, Halfon P, Fong A, Burnand B, Luthi JC, et al Coding algorithms for defining comorbidities in ICD-9-CM and ICD-10 administrative data. Med Care. 2005 11; 43(11):1130–9. 16224307 10.1097/01.mlr.0000182534.19832.83

[18] Schneeweiss S, Wang PS, Avorn J, Glynn RJ. Improved comorbidity adjustment for predicting mortality in Medicare populations. Health Serv Res. 2003 8;38(4):1103–20. 12968819 10.1111/1475-6773.00165 PMC1360935

[19] Middelburg RA, van de Watering LM, van der Bom JG. Blood transfusions: good or bad? Confounding by indication, an underestimated problem in clinical transfusion research. Transfusion. 2010 6;50(6):1181–3. 10.1111/j.1537-2995.2010.02675.x 20456689

[20] Zhang B, Wright AA, Huskamp HA, Nilsson ME, Maciejewski ML, Earle CC, et al Health care costs in the last week of life: associations with end-of-life conversations. Arch Intern Med. 2009 3 9;169(5):480–8. 10.1001/archinternmed.2008.587 19273778 PMC2862687

[21] Fergusson DA, Hébert PC, Mazer CD, Fremes S, MacAdams C, Murkin JM, et al A comparison of aprotinin and lysine analogues in high-risk cardiac surgery. N Engl J Med. 2008 5 29;358(22):2319–31. 10.1056/NEJMoa0802395 18480196

[22] Misset B, Nakache D, Vesin A, Darmon M, Garrouste-Orgeas M, Mourvillier B, et al Reliability of diagnostic coding in intensive care patients. Crit Care. 2008;12:R95 10.1186/cc6969 18664267 PMC2575581

[23] Como JJ, Dutton RP, Scalea TM, Edelman BB, Hess JR. Blood transfusion rates in the care of acute trauma. Transfusion. 2004 6;44(6):809–13. 15157244 10.1111/j.1537-2995.2004.03409.x

